# Fukushima-derived radionuclides in sediments of the Japanese Pacific Ocean coast and various Japanese water samples (seawater, tap water, and coolant water of Fukushima Daiichi reactor unit 5)

**DOI:** 10.1007/s10967-015-4386-9

**Published:** 2015-08-23

**Authors:** Katsumi Shozugawa, Beate Riebe, Clemens Walther, Alexander Brandl, Georg Steinhauser

**Affiliations:** Graduate School of Arts and Sciences, The University of Tokyo, Meguro-Ku, Tokyo, Japan; Institute of Radioecology and Radiation Protection, Leibniz Universität Hannover, Herrenhäuser Straße 2, 30419 Hannover, Germany; Environmental and Radiological Health Sciences, Colorado State University, 1618 Campus Delivery, Fort Collins, CO 80523 USA

**Keywords:** Fukushima, Environmental radioactivity, Pacific Ocean, Cesium, ^129^I, ^90^Sr, ^3^H

## Abstract

We investigated Ocean sediments and seawater from inside the Fukushima exclusion zone and found radiocesium (^134^Cs and ^137^Cs) up to 800 Bq kg^−1^ as well as ^90^Sr up to 5.6 Bq kg^−1^. This is one of the first reports on radiostrontium in sea sediments from the Fukushima exclusion zone. Seawater exhibited contamination levels up to 5.3 Bq kg^−1^ radiocesium. Tap water from Tokyo from weeks after the accident exhibited detectable but harmless activities of radiocesium (well below the regulatory limit). Analysis of the Unit 5 reactor coolant (finding only ^3^H and even low ^129^I) leads to the conclusion that the purification techniques for reactor coolant employed at Fukushima Daiichi are very effective.

## Introduction

Following the earthquake on March 11, 2011, a gigantic tsunami destroyed the cooling systems of Fukushima Daiichi nuclear power plant (NPP)(Japan) and caused partial melt-down of 3 reactor cores. In the course of the nuclear accident a total of 520 PBq (excl. noble gases) were released to the atmosphere [1]. More than 99 % of the released substances were radionuclides of Kr, Te, I, Xe, and Cs. These nuclides have been monitored globally in air [2, 3]. Less volatile radionuclides such as radiostrontium [4, 5], plutonium [6–8] or radionuclides that are difficult to measure, such as ^3^H [9, 10], ^135^Cs [11] or ^35^S [12] have been monitored much less frequently [13]. In any case, at least 80 % of the airborne radionuclides were transported offshore by the wind [14, 15], and a yet unknown amount has leaked directly into the Pacific Ocean. Although several studies have addressed the impact of the accident on the Pacific Ocean and its organisms [16–18], our knowledge on the impact of the Fukushima nuclear accident on the marine environment is yet far from complete.

In the current study, we investigated several interesting and unique sample materials, many of which were taken directly after the accident and/or inside the “exclusion zone” around the crippled reactors. Target nuclides were radiocesium [^134^Cs (*T*_1/2_ = 2.1 years) and ^137^Cs (*T*_1/2_ = 30.2 years)] as well as the understudied nuclides ^90^Sr (*T*_1/2_ = 28.6 years) and ^3^H (*T*_1/2_ = 12.3 years). For two samples, also ^129^I analysis was performed. It is a long-lived (*T*_1/2_ = 15.7 million years) fission product that can be used as an (environmental) tracer nuclide [19] that is also useful for retrospective dosimetry after the decay of short-lived ^131^I (*T*_1/2_ = 8 days) [20] which accumulates in the thyroid [21] and is mostly responsible for thyroid cancer cases after Chernobyl.

## Materials and methods

Environmental materials analyzed in this study included sediments and seawater from the Japanese Pacific Ocean coast inside the exclusion zone, tap water from Tokyo, seawater from Hawaii as well as a sample of the reactor coolant water of the undamaged Unit 5 of Fukushima Daiichi NPP. The coolant was investigated also for ^129^I to scrutinize the overall efficiency of the techniques currently employed for radionuclide removal from water at the Fukushima Daiichi NPP; most fission and activation products are cations that can be removed using natural or artificial cation exchanger. Iodine, however, is usually anionic (iodide or iodate) and thus more challenging to remove. A summary of all samples, the exact location and date of sampling, distance to the Fukushima Daiichi NPP as well as target nuclides is given in Table 1.

**Table 1:** Samples investigated in this study

Sample type	Sample no./code	Sample location	Sampling date	Distance to FDNPP (km)	Radionuclides analyzed
Ocean sediment	Sed 1	37.4040258; 141.033522	2011-12-20	1.95	^90^Sr, ^134,137^Cs
Ocean sediment	Sed 2	37.478988; 141.039604	2011-12-20	6.54	^90^Sr, ^134,137^Cs
Ocean sediment	Sed 3	37.567682; 141.026177	2011-12-20	16.1	^90^Sr, ^134,137^Cs
Ocean sediment	Sed 4	37.21161; 141.005861	2011-12-20	23.5	^90^Sr, ^134,137^Cs
Ocean sediment	Sed 5	37.13266; 140.998351	2011-12-20	32.2	^90^Sr, ^134,137^Cs
Ocean water	OW 1	37.4040258; 141.033522	2011-12-20	1.95	^3^H, ^90^Sr, ^134,137^Cs
Ocean water	OW 2	37.478988; 141.039604	2011-12-20	6.54	^3^H, ^90^Sr, ^134,137^Cs
Ocean water	OW 3	37.567682; 141.026177	2011-12-20	16.1	^3^H, ^90^Sr, ^134,137^Cs
Ocean water	OW 4	37.13266; 140.998351	2011-12-20	23.5	^3^H, ^90^Sr, ^134,137^Cs
Ocean water	OW 5	37.21161; 141.005861	2011-12-20	32.2	^3^H, ^90^Sr, ^134,137^Cs
Tap water	TW 6	Tokyo, Japan	2011-03-21	225	^3^H, ^90^Sr, ^134,137^Cs
Tap water	TW 7	Tokyo, Japan	2011-03-24	225	^3^H, ^90^Sr, ^134,137^Cs
Tap water	TW 8	Tokyo, Japan	2011-03-27	225	^3^H, ^90^Sr, ^134,137^Cs
Tap water	TW 9	Tokyo, Japan	2011-03-31	225	^3^H, ^90^Sr, ^134,137^Cs
Hawaii Ocean water	HI W	Kona, Big Island, HI	2012-03-31	6350	^129^I
Reactor unit 5 coolant water	CW #5	37.428526; 141.032841	2011-10-22	<0.75	^3^H, ^90^Sr, ^129^I, ^134,137^Cs

**Fig. 1:**
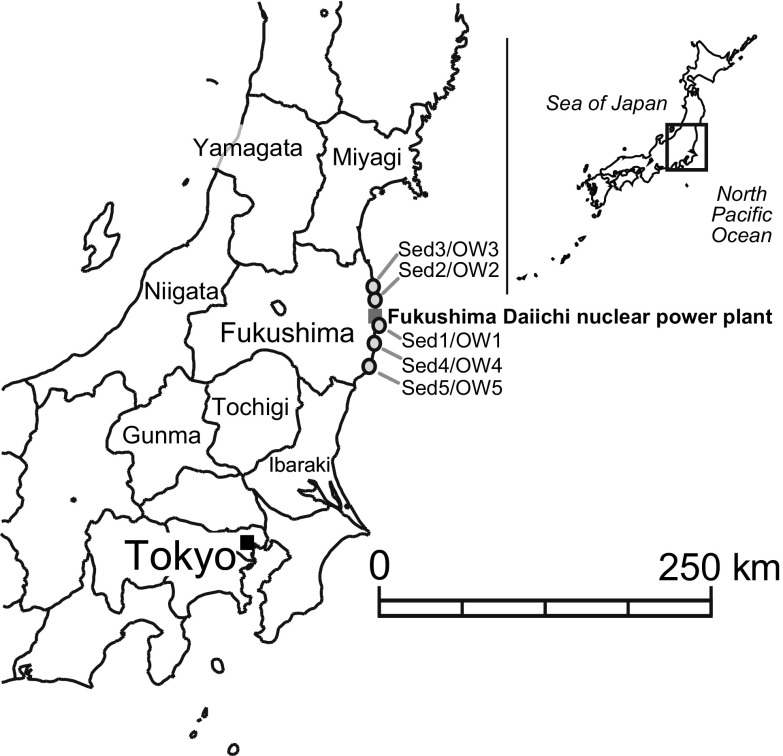
Geographical setting of the sampling sites in Japan

A map of the sample locations (excluding the seawater sample from Hawaii), is given in Fig. 1.

Gamma measurements for ^134^Cs and ^137^Cs were conducted with an ORTEC™ 364 cm^3^ HPGe detector with a 0.76 mm Be window (2.32 keV resolution at the 1332 keV ^60^Co peak; 87.4 % relative efficiency). The samples were weighed into 60 or 125 mL polypropylene containers (typically 100 mL for water samples; 70 g for sediment samples) that were placed on top of the detector. The samples were measured for at least 24 h. Detector efficiency calibration was done with an Eckert and Ziegler^®^ multinuclide standard solution that was diluted in 2 M HCl (aqueous samples) or dispersed in quartz sand (sediment samples) (see [22]). Tritium (^3^H) and ^90^Sr was conducted with liquid scintillation counting (LSC). The LSC measurements were performed using a LabLogic 300SL Liquid Scintillation Counter with TDCR Technology (Hidex, Mustionkatu 2, FIN-20750 Turku, Finland). The user interface software is provided by MikroWin™ (Mikrotek Laborsysteme GmbH, Olper Strasse 35, D-51491 Overath, Germany). Instrument output was transferred to a vendor-provided PC-application spreadsheet with macros where the raw data could be analyzed and a graphic routine provided for convenient data visualization [23].

For radiocesium measurements, the sediment samples were dried at 100 °C, weighed and filled into calibrated geometries at the gamma detector station at Colorado State University. Water samples were weighed and measured for gamma emitters without further pre-treatment.

Pure beta emitters such as ^90^Sr or ^3^H require chemical separation and purification prior to beta counting in the LSC. Water samples were carefully distilled to dryness; the distillate was mixed with Ultima Gold™ low level tritium (LLT) scintillation cocktail for measurement. Ocean sediments were treated with nitric acid for Sr leaching and purified using the Eichrom™ SR resin (2 mL cartridges), as described elsewhere [4]. In brief, the separation process involved weighing of the dried sand into a flask, addition of 8 mL HNO_3_ (8 M), 2 mL H_2_O_2_ (30 %), 1 ml Sr carrier solution (*c*_Sr_ = 1.2 mg mL^−1^), and 3 mL of concentrated HNO_3_ (70 %). This solution was boiled under reflux for 30 min and then filtered through a paper filter. The filtrate was loaded onto preconditioned (with 8 M HNO_3_) SR resin cartridges. The flask was rinsed with 3 × 2 mL HNO_3_ (8 M). The resin was then rinsed with 10 × 1 mL of a mixture of HNO_3_ (3 M) and oxalic acid (0.05 M). Then the Sr fraction was eluted with 0.01 M HNO_3_, which we found to be sufficiently acidic to not eluate any radiolead from the column. Water samples (50 mL) were either brought to 8 M HNO_3_ by addition of equal amounts of 16 m HNO_3_, or the solid residue after tritium distillation was taken up in 8 M HNO_3_. Since the natural Sr content in water is much lower than from leached sediments, the amount of Sr carrier was increased to 1.7 mg. The loading, rinsing and eluting processes were conducted as described above.

We found that the acidic eluate (0.01 M HNO_3_) does not mix well with the Ultima Gold™ LSC cocktail. In order to remove the acid, the eluate was evaporated to almost dryness and then taken up in 1 mL H_2_O again. This step was repeated 10 times. Then, the Sr fraction was transferred to LSC vials and the flask was rinsed with 4 × 0.5 mL H_2_O. Finally 18 mL of scintillation cocktail were added.

Addition of HNO_3_ to sediments should be performed carefully as the carbonate fraction of the sand will vigorously decompose under formation of CO_2_ gas which could cause the overflow of the flask with acidic foam. In our case, the sediments proved to have a very low carbonate content. Also organic substances may react violently upon exposure to the highly oxidizing HNO_3_/H_2_O_2_ mixture, especially when heated. If in doubt, the use of protective measures (such as gloves, face shields) is recommended [24–26].

For the accelerator mass spectrometry (AMS) measurements of ^129^I, iodine was separated from the matrix in two steps. First, Woodward iodine carrier was added and all the iodine was transferred into iodide. All iodine species were oxidized with Ca(OCl)_2_ to iodate and afterwards reduced with NH_3_OHCl and NaHSO_3_ to iodide. The second step consisted in an ion exchange separation using a DOWEX^®^ 1 × 8 analytical grade ion exchange resin, which was preconditioned with KNO_3_. After rinsing the ion exchange columns with high purity water and a 0.5 mol L^−1^ KNO_3_ solution, the iodine was eluted with concentrated potassium nitrate solution (2.25 mol L^−1^). The iodine was precipitated as AgI, which was dried, mixed with silver powder (AgI:Ag 1:4 by weight), and pressed into titanium targets for AMS measurement. Measurements were performed with low-energy accelerator mass spectrometry (AMS) at the 0.5 MV TANDY facility at ETH Zurich, Switzerland.

For the ICP-MS measurement of ^127^I no iodine matrix separation was necessary. The samples were diluted and tetramethylammoniumhydroxide (TMAH) was added, which causes hydrolysis of any organic compounds and reduces the redox potential. The stability of I^–^ increases in the sample relative to I_2_ and IO_3_^−^. Moreover, iodide and iodate have not significantly different sensitivities in the ICP-MS measurements. A Thermo X7 (Thermo Fisher Scientific) was employed for the detection and quantification of stable ^127^I [27].

## Results and discussion

Results of the radionuclide analysis are summarized in Table 2. Tritium, ^90^Sr, ^134^Cs, and ^137^Cs were obtained by radiometric means, ^129^I by AMS. Please note that the seawater sample from Hawaii was only analyzed for ^129^I, as radiometric methods were not deemed successful for the presumably very low contamination levels.

**Table 2:** Results of the radionuclide analyses on Pacific Ocean sediments and water samples in Bq·kg^−1^ at the time of sampling

Sample no.	^3^H	^90^Sr	^129^I	^134^Cs	^137^Cs
Sed 1	N/A	5.4 ± 0.8	N/A	357 ± 20	436 ± 11
Sed 2	N/A	5.6 ± 0.8	N/A	142 ± 12	156 ± 5
Sed 3	N/A	3.8 ± 0.6	N/A	19 ± 6	25.3 ± 2.4
Sed 4	N/A	2.8 ± 0.4	N/A	177 ± 2	229 ± 1
Sed 5	N/A	3.2 ± 0.5	N/A	102 ± 2	134 ± 2
OW 1	<0.1	<0.4	N/A	2.5 ± 0.7	2.8 ± 0.3
OW 2	<0.1	<0.4	N/A	<1.3	1.2 ± 0.4
OW 3	<0.1	<0.4	N/A	<2.9	<0.8
OW 4	<0.1	<1	N/A	1.8 ± 0.6	<0.4
OW 5	<0.1	<0.4	N/A	1.7 ± 0.6	1.1 ± 0.3
TW 6	<0.1	<0.4	N/A	< 0.32	1.6 ± 0.6
TW 7	<0.1	<0.4	N/A	1.52 ± 0.40	1.69 ± 0.14
TW 8	<0.1	<0.4	N/A	3.71 ± 0.35	4.27 ± 0.13
TW 9	<0.1	<0.4	N/A	1.57 ± 0.39	0.95 ± 0.25
HI W	N/A	N/A	2.37E−8 ± 1.9E−9*	N/A	N/A
CW #5	2260 ± 230	< 0.4	1.29E−6 ± 3.4E−8**	<4	<1.3

Activity concentrations for sediment samples are given per dry mass. Uncertainties of radiometric measurements are due to counting statistics

*N/A* not analyzed

* ^129^I/^127^I ratio 1.10E−10

** ^129^I/^127^I ratio 2.63E−8

### Ocean sediments

**Fig. 2:**
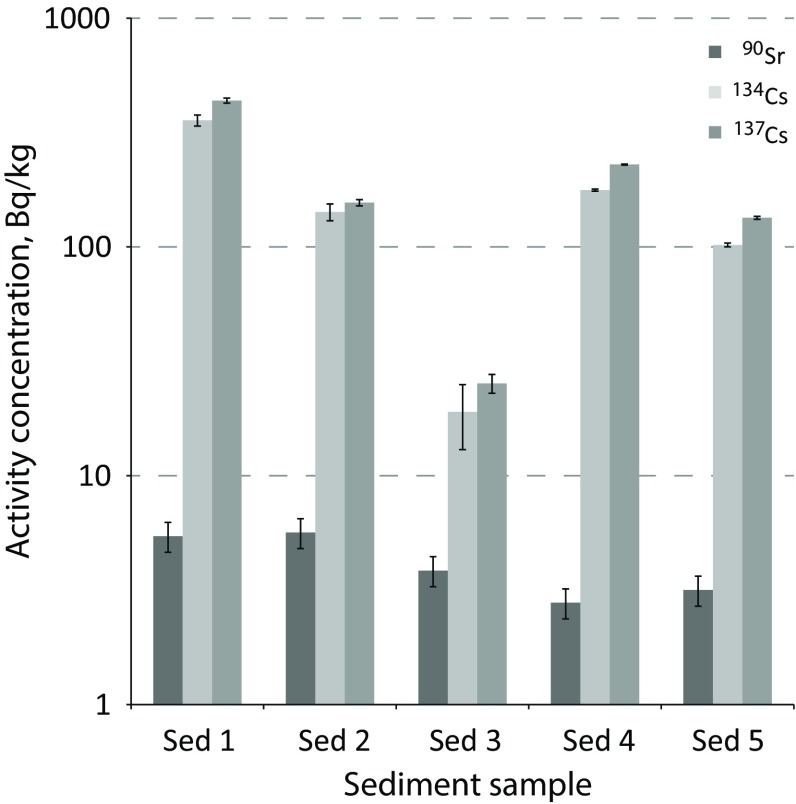
Activity concentrations of ^134^Cs, ^137^Cs and ^90^Sr in Fukushima Daiichi exclusion zone Pacific Ocean sediments (dry mass)

As expected, Pacific Ocean sediments from inside the exclusion zone exhibited the highest contamination levels. The correlation between radiostrontium and ^134^Cs as well as ^137^Cs is illustrated in Fig. 2.

Although a slight distance dependency can be observed (at least sample Sed 1 that was taken closest to the Fukushima Daiichi NPP exhibited the highest activity concentration), it was surprising to note that sample Sed 3 (taken 16.1 km north of the NPP) exhibited the lowest activities. It is also interesting that the radiocesium activity concentrations fluctuate by more than an order of magnitude within the samples, the ^90^Sr activity concentrations, however, only by a factor of 2. The ^90^Sr/^137^Cs activity ratios (where calculable) were 0.012 ± 0.002 (Sed 1), 0.036 ± 0.005 (Sed 2), 0.15 ± 0.03 (Sed 3), 0.012 ± 0.002 (Sed 4), and 0.024 ± 0.004 (Sed 5). It is unclear why Sed 3 has a ten times higher ^90^Sr/^137^Cs ratio than the other sediments (which fluctuate within a factor of 3). This odd ratio is not so much due to a high ^90^Sr concentration, which is in the range of the other samples, but rather due to an exceptionally low ^137^Cs concentration. The reason for this anomaly (which has been observed in similar manner in terrestrial environmental samples from north of the NPP [4] ) is yet unclear. Although, from an radioecological point of view, sediment is not comparable to food, the high ^90^Sr/^137^Cs ratio of 0.15 challenges the governmental assumption for the regulatory limits in food, namely a constant ^90^Sr/^137^Cs ratio in food of ≤0.1 [28]. Seafood organisms exposed to high-^90^Sr effluents may exhibit higher ^90^Sr concentrations than covered by this governmental assumption.

The ^134^Cs/^137^Cs activity ratios are reliable source identifiers [29]. The average ^134^Cs/^137^Cs ratio at the time of the accident (March 11, 2011) was found to be 0.98 [28]. Due to the shorter half-life of ^134^Cs, this average ratio has been calculated to have gone down to 0.77 by the time of sampling (Dec 20, 2011). The ratios found were 0.81 ± 0.05 (Sed 1), 0.91 ± 0.08 (Sed 2), 0.75 ± 0.25 (Sed 3), 0.77 ± 0.01 (Sed 4), and 0.76 ± 0.02 (Sed 5). Sed 2 seems to exhibit a somewhat higher ^134^Cs/^137^Cs ratio than the other sediments, however, with the given uncertainty margins, this outlier seems not significant. It would be interesting to study the ^135^Cs/^137^Cs ratio [11] to possibly identify the source of these contaminations.

Other studies [30] for radiocesium in sea sediments found up to 250 Bq kg^−1^-dry in Sendai (north of the Fukushima Daiichi NPP), which is naturally somewhat lower than the levels reported in this study, but in good agreement with our results. Our findings are also in good agreement with the activity concentrations reported by Yamamoto et al. [31]. A study on sediments from the Sea of Japan naturally found much lower radiocesium activity concentrations (0.25 Bq kg^−1^^137^Cs and 0.15 Bq kg^−1^^134^Cs in the Sado Basin) [32].

For comparison, it may be interesting to note that the activity levels found in these Ocean Sediments are roughly comparable to the radiocesium inventory in soil in Central Europe, e.g., in Austria [22], which is still significantly polluted from the Chernobyl accident and the fallout from 20^th^ century’s atmospheric explosions.

### Seawater

With only small volumes available for analysis, it was not expected to detect any radionuclides. However, low activities of radiocesium were found (with high uncertainties), as summarized in Table 2. Given the high uncertainties ^134^Cs/^137^Cs ratios are less meaningful; they are 0.89 ± 0.3 (OW 1), <1.08 (OW2), >4.5 (OW4), and 1.5 ± 0.7 (OW5). In any case, our findings for seawater (up to 2.8 Bq kg^−1^^137^Cs and 2.5 Bq kg^−1^^134^Cs) are in good agreement with previously reported data [30] for seawater.

No tritium was detected in any of the seawater samples. The ^129^I activity concentration in the seawater sample from Hawaii from 2012 (2.37 × 10^−8^ ± 1.9 × 10^−9^; ^129^I/^127^I ratio 1.10 × 10^−10^) are in the range of the pre-Fukushima background reported by Stan-Sion et al. [33].

### Tap water

Tap water from Tokyo exhibited contamination levels that were of no health concern (up to 4.3 Bq kg^−1^^137^Cs). The activity concentrations found in this study are in good agreement with previously reported tap water results [28, 29]. The radiocesium activity concentrations were clearly below the regulatory limit for radiocesium in tap water of 200 Bq kg^−1^. Previous studies [28] showed that ^131^I was probably also present in Tokyo’s tap water at the time of sampling; however, the measurements of this study were performed too late for the detection of this short-lived radionuclide. Strontium-90 and tritium could not be detected in tap water because of too low sample volumes and the limited detection limit of the LSC, respectively.

The ^134^Cs/^137^Cs activity ratios slightly deviated from the average ratio [28] of 0.98 at the time of the accident (0.97–0.96 at the time of sampling would have suggested): we found ratios of <0.2 (TW6), 0.90 ± 0.25 (TW7), 0.87 ± 0.09 (TW8), and 1.7 ± 0.6 (TW9). Like TW6, TW7, and TW8, also the ratios found in Tokyo tap water in a previous study [28] were consistently lower than the average; only TW9 exhibited a higher ratio, which raises the question of the source of these contaminations.

### Reactor coolant

Without a doubt, the sample of reactor coolant was the most “unusual” sample of this study. The analysis of this sample allows the investigation of the performance of the water purification techniques currently employed at the Fukushima Daiichi NPP. Purification is performed in multiple steps, including the application of charcoal and zeolithe as adsorbent materials as well as reverse osmosis techniques. This purification method proves to be extremely efficient: No radiocesium and no radiostrontium could be detected. Also, the content of ^129^I is surprisingly low; it is just two orders of magnitude higher than 2012 Pacific Ocean water from Hawaii (see Table 2). By far the dominant activity in the reactor coolant is, as expected, tritium (HTO) that cannot be removed from water by chemical means.

## Conclusions

The sea sediments investigated in this study were found to be contaminated with radiocesium (up to almost 800 Bq kg^−1^) and radiostrontium (up to 5.6 Bq kg^−1^). This is one of the first reports on radiostrontium in sea sediments from the Fukushima exclusion zone. Seawater exhibited radiocesium contamination levels up to 5.3 Bq kg^−1^, which is also in agreement with previous findings. Tap water from Tokyo weeks after the accident exhibited detectable but harmless activities of radiocesium (well below the regulatory limit). The investigation of the reactor coolant (finding only ^3^H and even low ^129^I) leads to the conclusion that the purification techniques for reactor coolant employed at Fukushima Daiichi are very effective.
